# iTRAQ-Based Quantitative Proteomic Analysis of a Toxigenic Dinoflagellate *Alexandrium catenella* at Different Stages of Toxin Biosynthesis during the Cell Cycle

**DOI:** 10.3390/md16120491

**Published:** 2018-12-07

**Authors:** Shu-Fei Zhang, Yong Zhang, Lin Lin, Da-Zhi Wang

**Affiliations:** 1 State Key Laboratory of Marine Environmental Science, College of the Environment and Ecology, Xiamen University, Xiamen 361000, China; zhangshufei@scsfri.ac.cn (S.-F.Z.); foryzhy@gmail.com (Y.Z.); linlin1982@xmu.edu.cn (L.L.); 2 Guangdong Provincial Key Laboratory of Fishery Ecology and Environment, South China Sea Fisheries Research Institute, Chinese Academy of Fishery Sciences, Guangzhou 510300, China; 3 School of Environmental Science and Engineering, Xiamen University Tan KahKee College, Zhangzhou 363105, China; 4 Key Laboratory of Marine Ecology & Environmental Sciences, Institute of Oceanology, Chinese Academy of Sciences, Qingdao 266071, China

**Keywords:** dinoflagellates, *Alexandrium catenella*, paralytic shellfish toxins, cell cycle, toxin biosynthesis, quantitative proteomics, iTRAQ

## Abstract

Paralytic shellfish toxins (PSTs) are a group of potent neurotoxic alkaloids that are produced mainly by marine dinoflagellates. PST biosynthesis in dinoflagellates is a discontinuous process that is coupled to the cell cycle. However, little is known about the molecular mechanism underlying this association. Here, we compared global protein expression profiles of a toxigenic dinoflagellate, *Alexandrium catenella*, collected at four different stages of toxin biosynthesis during the cell cycle, using an isobaric tags for relative and absolute quantification (iTRAQ)-based quantitative proteomic approach. The results showed that toxin biosynthesis occurred mainly in the G1 phase, especially the late G1 phase. In total, 7232 proteins were confidently identified, and 210 proteins exhibited differential expression among the four stages. Proteins involved in protein translation and photosynthetic pigment biosynthesis were significantly upregulated during toxin biosynthesis, indicating close associations among the three processes. Nine toxin-related proteins were detected, and two core toxin biosynthesis proteins, namely, sxtA and sxtI, were identified for the first time in dinoflagellates. Among these proteins, sxtI and ompR were significantly downregulated when toxin biosynthesis stopped, indicating that they played important roles in the regulation of PST biosynthesis. Our study provides new insights into toxin biosynthesis in marine dinoflagellates: nitrogen balance among different biological processes regulates toxin biosynthesis, and that glutamate might play a key modulatory role.

## 1. Introduction

Paralytic shellfish toxins (PSTs) are a group of neurotoxic alkaloids that selectively block voltage-gated sodium channels in excitable cells, resulting in approximately 2000 incidents of paralytic shellfish poisoning (PSP) annually worldwide [1]. In the past few decades, PSP has caused increasing concern due to the global increase in the frequency, intensity and geographic distribution of PST-producing algal blooms [1,2].

Recently, the PST biosynthetic pathway has been unveiled in several species of cyanobacteria, which are another important PST-producing group in addition to dinoflagellates [3,4,5,6]. A group of core genes (*sxtA*, *sxtG*, *sxtB*, *sxtD*, *sxtS*, *sxtU*, *sxtH/T* and *sxtI*) that are directly involved in toxin biosynthesis, tailor genes (*sxtL*, *sxtN*, *sxtX*) that participate in toxin transformation and some additional genes that are responsible for toxin transportation and regulation are characterized, and several toxin-related proteins are also identified in cyanobacteria [3,7]. In contrast, the toxin biosynthetic pathway in dinoflagellates remains unclear, although the pathway is believed to be similar to that in cyanobacteria [8]. To date, some putative homologs of cyanobacterial toxin genes and proteins have been identified in dinoflagellates [9,10]; however, only *sxtA* and *sxtG*, which participate in the first two biosynthetic steps, have been well characterized [11,12]. The *sxtA* gene of dinoflagellates has two isoforms: the long isoform contains all the *sxtA1*–*A4* catalytic domains, while the short isoform contains only domains *sxtA1*–*A3*, not *sxtA4*, which is essential for PST biosynthesis [11,13,14,15].

Studies have shown that PST production in toxigenic dinoflagellates is a discontinuous process coupled to a restricted time frame of the cell cycle. PST biosynthesis in *Alexandrium fundyense* is regulated by light and mainly occurs in the G1 phase [16,17,18]. However, PST biosynthesis in *Alexandrium tamarense* occurs in the early stage of the S phase [19]. Similarly, toxin synthesis in *Alexandrium catenella* does not occur in the G1 phase but in the S phase or G2/M phase of the cell cycle [20,21]. Although the toxin-producing periods of different species and strains are not consistent, all these results indicate that the production process in dinoflagellates is regulated by the cell cycle and occurs during a specific period of the cell cycle. Further studies have revealed that three genes related to the biosynthesis of PST precursors are upregulated during toxin production in *A. fundyense* [21], and the expression of nine proteins that are potentially involved in toxin biosynthesis in *A. catenella* vary significantly at different stages of toxin biosynthesis [22]. However, the molecular mechanism underlying the association between the cell cycle and toxin synthesis remains unclear.

In our recent studies, several genes and cellular processes potentially involved in PST biosynthesis are identified, and toxin production is found to be regulated translationally or post-translationally [10,15,23,24]. In this study, we compared the global protein expression profiles of *A. catenella* at four different toxin biosynthesis stages in the cell cycle using an isobaric tags for relative and absolute quantification (iTRAQ)-based quantitative proteomic approach. Our goal was to mine toxin-related proteins and to identify molecular processes associated with PST biosynthesis at the protein level.

## 2. Results

### 2.1. Cell Cycle Distribution and Toxin Content Variation

**Figure 1:**
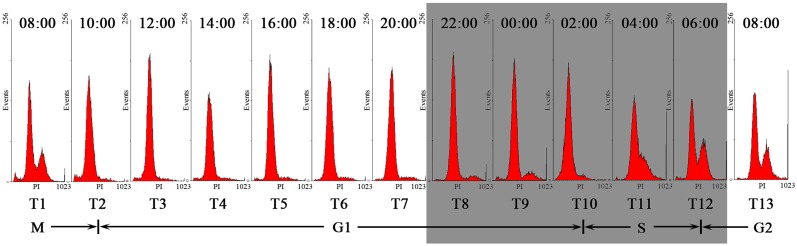
Flow cytometric analysis of ACHK-T during the cell cycle. X-axis: Relative DNA content; Y-axis: Cell number. The gray box represents the dark period.

Variations in the DNA histogram indicated that the toxic *A. catenella* strain, ACHK-T, completed a cell cycle within one day (Figure 1). At 10:00, the cells entered the G1 phase, which lasted for 16 h, until 02:00 the next day. Then, the cells entered the S phase between 02:00 and 06:00, after which, the cells entered the G2/M phase and completed cell division by 10:00.

**Figure 2:**
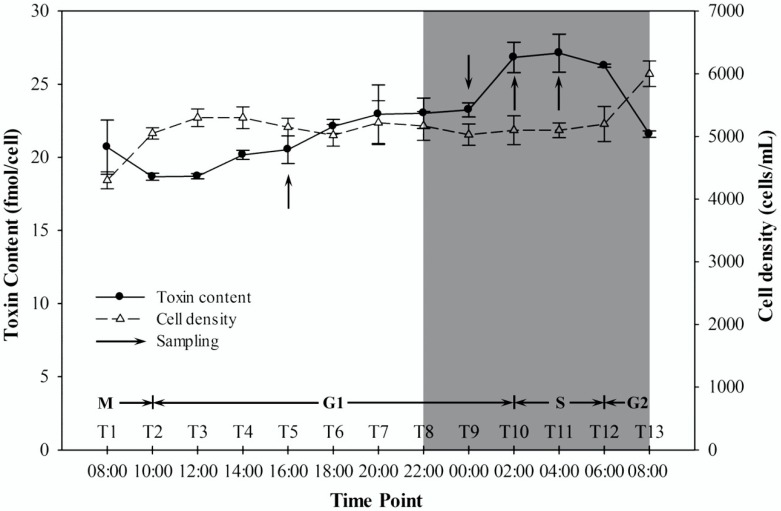
Variations of cell density and intracellular toxin content during the cell cycle. Both values are reported as the means of biological triplicates with standard deviations. The gray box represents the dark period.

The cell density remained stable during the cell cycle except for a sharp increase in the G2/M phase (Figure 2). The toxin content increased slowly during the light period (the early and middle G1 phases) but increased sharply between 00:00 to 02:00, the last 2 h of the G1 phase. Subsequently, the toxin content remained at the highest level for approximately 4 h and decreased sharply when cell division started at 06:00 (Figure 2).

### 2.2. Proteome Overview and Protein Annotation

Overall, 71,413 of the total 345,182 output spectra were matched to 22,299 peptides with an approximately 20.7% spectrum utilization rate. Using the Mascot search engine (version 2.3.02; Matrix Science, London, United Kingdom), 7232 proteins were identified from 19,227 unique peptides that collectively matched 51,904 unique spectra. All of the proteins identified were annotated using the Kyoto Encyclopedia of Genes and Genomes (KEGG) and National Center for Biotechnology Information nonredundant protein sequences (NCBInr) databases (Supplementary Table S1).

**Figure 3:**
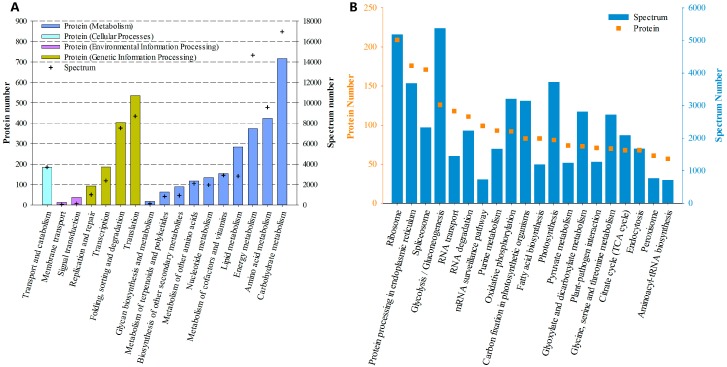
Functional distribution of proteins based on Kyoto Encyclopedia of Genes and Genomes (KEGG) annotation. (**A**) Distribution based on the secondary hierarchy. (**B**) Top 20 abundant pathways (according to protein number) based on the tertiary hierarchy.

Based on the KEGG categories, proteins were annotated into 17 groups. “Carbohydrate metabolism”, “Amino acid metabolism” and “Energy metabolism” were the top three categories under “Metabolism”, while “Translation” and “Folding, sorting and degradation” were the dominant categories under “Genetic Information Processing” (Figure 3A). Further tertiary hierarchical classification showed that “Ribosome”, “Protein processing in endoplasmic reticulum” and “Spliceosome” were the most frequently detected pathways (Figure 3B).

To further annotate the protein functions, all the proteins were subjected to NCBInr database analysis. With a criterion of e-value ≤ 1 × 10^−5^, a total of 4924 proteins were successfully assigned with diverse functional annotations (Supplementary Table S1). The most abundant proteins (according to spectrum number) were associated with photosynthesis, such as the peridinin-chl a protein, chlorophyll a/c-binding protein and ribulose 1,5-bisphosphate carboxylase oxygenase. Moreover, the luciferin-binding protein, which is involved in bioluminescence, was also present in the list of the 10 most abundant proteins.

### 2.3. Differentially Expressed Proteins

**Figure 4:**
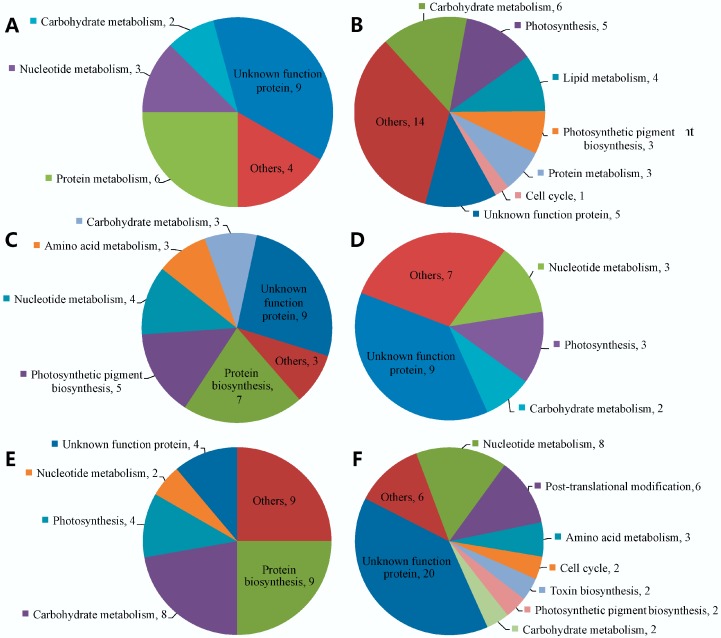
Functional distribution of differentially expressed proteins. (**A**) Upregulated proteins in T5 vs. T9. (**B**) Downregulated proteins in T5 vs. T9. (**C**) Upregulated proteins in T9 vs. T10. (**D**) Downregulated proteins in T9 vs. T10. (**E**) Upregulated proteins in T10 vs. T11. (**F**) Downregulated proteins in T10 vs. T11.

Of the 7232 proteins identified, 65 proteins in T5 vs. T9, 58 proteins in T9 vs. T10 and 87 proteins in T10 vs. T11 exhibited differential expression. In T5 vs. T9, 24 proteins were upregulated and 41 proteins were downregulated; in T9 vs. T10, 34 proteins were upregulated and 24 proteins were downregulated; and in T10 vs. T11, 36 proteins were upregulated and 51 proteins were downregulated. Functional classification of these proteins based on KEGG and NCBInr annotation is presented in Figure 4 and Supplementary Table S2.

Quantitative analysis showed that proteins involved in protein translation were upregulated in T9 vs. T10, which was the toxin biosynthesis period. These proteins included ribosomal protein subunits, translation initiation factors, elongation factors and some aminoacyl-tRNA synthetases. Proteins participating in photosynthetic pigment biosynthesis were downregulated in T5 vs. T9 and T10 vs. T11 but upregulated significantly in T9 vs. T10. Moreover, the cell cycle-related protein E3 SUMO-protein ligase RanBP2 was downregulated in T5 vs. T9, and two other proteins, namely, kinesin family member 11 and cell division control protein 47, were downregulated in T10 vs. T11. However, notably, a large number of differentially expressed proteins were annotated with unknown functions, especially in T9 vs. T10, in which the toxin content increased sharply. These proteins might be involved in toxin biosynthesis or might participate in other biological processes associated with toxin production.

### 2.4. Toxin-Related Proteins

With a cutoff e-value ≤ 1 × 10^−5^, a total of 43 homologs of nine toxin-related proteins were identified in *A. catenella*, including sxtA, sxtG, sxtH, sxtI, sxtO, sxtT, sxtU, sxtZ and ompR (Table 1). Among these proteins, sxtA and sxtI were detected in dinoflagellates for the first time. Quantitative analysis showed that the expression levels of sxtI and ompR were significantly downregulated in T10 vs. T11, while those of other toxin-related proteins remained relatively stable.

## 3. Discussion

In this study, we compared the global protein expression profiles of ACHK-T at four different toxin biosynthesis stages using an iTRAQ-based proteomic approach. A total of 210 proteins exhibited differential expression in T5 vs. T9, T9 vs. T10 and T10 vs. T11. These proteins are involved in various biological processes, such as PST biosynthesis, protein translation and photosynthetic pigment biosynthesis, and some of these proteins exhibited different variations in these three pairwise comparisons, which might be associated with toxin production.

### 3.1. Toxin Biosynthesis

High-performance liquid chromatography (HPLC) analysis showed that the toxin content exhibited periodic variation: toxin content increased rapidly in the late G1 phase and decreased sharply when the cells entered the G2/M phase. This variation was similar to that observed in our previous study [24] but differed from the results of previous studies on *Alexandrium* [16,18,20,22], which might be caused by species [16,18] or strain specific differences [20,22]. In addition, different culture conditions such as temperature or irradiance might be other possibilities resulting in the difference [16,18,20].

Nine toxin-related proteins were identified, and sxtA and sxtI were first detected in dinoflagellates. Among these proteins, sxtA is the key protein involved in the first step of the PST biosynthetic pathway [3,11]. In toxigenic dinoflagellates, the gene *sxtA* is transcribed into two types of transcripts: a long transcript containing all four domains (*sxtA1–sxtA4*), and a short transcript containing the first three domains but not *sxtA4* [11]. Several studies have revealed that the *sxtA4* domain performs a Claisen condensation reaction and is essential for PST production in dinoflagellates [9,15,25]. In our study, sxtA was identified as the long isoform (see Protein ID 6131 in Supplementary Table S1); therefore, the one unique peptide identified for this protein should belong to the sxtA4 domain. However, this protein was not quantified among the four different toxin production stages because of the presence of only one unique peptide. This finding indicated that the abundance of some proteins involved in PST production might be very low.

SxtI is another core protein that is directly involved in toxin production. This protein catalyzes the transfer of a carbamoyl group from carbamoyl phosphate to an intermediate product of PSTs [3]. Quantitative analysis showed that sxtI was significantly downregulated in T10 vs. T11 but varied nonsignificantly in T5 vs. T9 and T9 vs. T10 (Table 1). A similar expression pattern was also observed for ompR, a transcriptional regulator of the toxin biosynthetic pathway [3] (Table 1). Downregulation of these two proteins in T10 vs. T11 was consistent with the toxin biosynthesis pattern because the cells stopped toxin production during this period (Figure 2), and the variation in ompR level suggested that the regulation of toxin production in dinoflagellates might be similar to that in cyanobacteria. However, the expression of these two proteins varied nonsignificantly in T5 vs. T9 and T9 vs. T10, especially in T9 vs. T10, in which the toxin content increased sharply (Figure 2). Moreover, the expression of other toxin-related proteins varied nonsignificantly among the three pairwise comparisons. Previous studies reported that some important biological processes including toxin production are regulated at translational or post-translational level in dinoflagellates [25,26,27,28]. Therefore, our results indicated that the toxin biosynthetic process in dinoflagellates was more complex than that in cyanobacteria, and the PST biosynthesis might be regulated post-translationally rather than translationally.

### 3.2. Protein Translation

**Figure 5:**
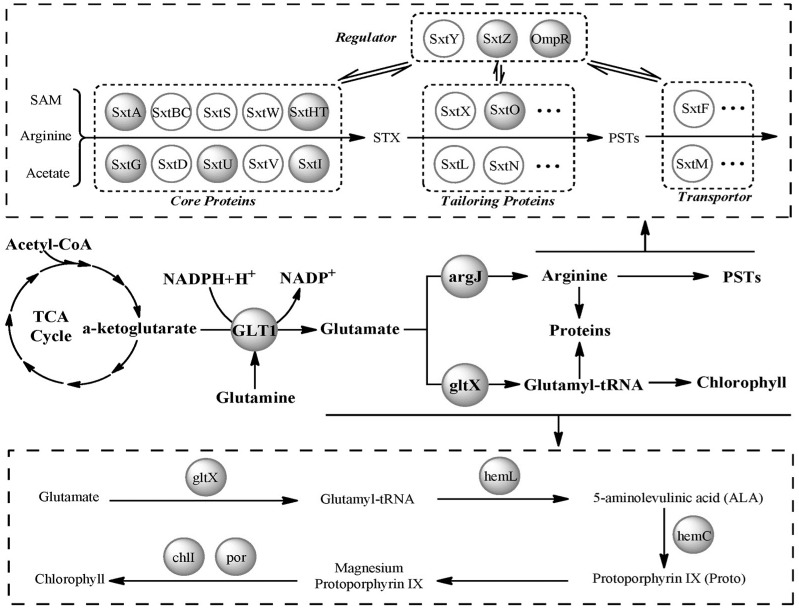
Relationships among paralytic shellfish toxin (PST) production, protein translation and chlorophyll biosynthesis. Dark circles represent the proteins identified in this study. The dashed box on top shows the putative PST biosynthesis pathway in dinoflagellates (modified from previous reports [4,9,32]), and the dashed box on the bottom summarizes the route of chlorophyll biosynthesis (modified from previous reports [33,34]). The abbreviations are: TCA: tricarboxylic acid cycle; gltX: glutamyl-tRNA synthetase; chlI: magnesium chelatase subunit I; por: protochlorophyllide reductase; hemL: oxygen-dependent protoporphyrinogen oxidase; argJ: glutamate N-acetyltransferase/amino acid N-acetyltransferase; GLT1: glutamate synthase (NADH).

In our study, a number of proteins involved in protein translation were significantly upregulated in T9 vs. T10, including one ribosomal protein, three translation factors that directly participate in the translation process, four aminoacyl-tRNA synthetases and three amino acid metabolism proteins that provide substrates for protein translation (Supplementary Table S2). The upregulation of these proteins suggested that the protein translation process was enhanced, which was coincident with PST production. A similar correlation between these two biological processes was also observed in *A. fundyense* [17]. It is postulated that PST production of dinoflagellates is a specific pathway integrated in the cellular nitrogen metabolism [17]. PST biosynthesis is indirectly modulated by protein biosynthesis and amino acid metabolism, and, as a common substrate, arginine acts as the link between these processes (Figure 5) [17,29,30]. Among the three amino acid metabolism proteins mentioned above, glutamate N-acetyltransferase/amino acid N-acetyltransferase is a key bifunctional protein that is involved in arginine biosynthesis, while glutamate synthase catalyzes the biosynthesis of glutamate, which is the substrate of arginine and glutamine—another nitrogen-enriched intermediate for the biosynthesis of many macromolecules [31]. Upregulation of these two proteins suggested that glutamate and arginine biosynthesis were enhanced, which provided enough substrate and intermediate for protein translation and toxin production during this period. Overall, our results supported the previous opinion that PST production might be a mechanism by which the balance of intracellular nitrogen metabolism is maintained in dinoflagellates.

### 3.3. Photosynthetic Pigment Biosynthesis

In marine dinoflagellates, chlorophyll and carotenoids are two major groups of photosynthetic pigments and play essential roles in photosynthetic light harvesting and energy transduction [35]. The biosynthetic process of chlorophyll can be divided into three steps (Figure 5): (i) the formation of 5-aminolevulinic acid (ALA), which is the rate limited and committed step of the whole pathway; (ii) the formation of protoporphyrin IX (Proto) from eight molecules of ALA; and (iii) the formation of chlorophyll in the magnesium branch [33,34]. Previous studies have revealed that the accumulation of photosynthetic pigments and the proteins involved in this process are adapted to the circadian clock or light–dark cycle [36,37]. Chlorophyll biosynthesis is restricted to the light period, and the proteins involved in this process are coordinately expressed [36,38,39]. In our study, the T9, T10 and T11 sampling points were in the dark period, and the T5 sampling point was in the light period. Among the differentially expressed proteins, glutamate-1-semialdehyde 2,1-aminomutase (hemL), hydroxymethylbilane synthase (hemC) and protochlorophyllide reductase (por), which participate in the three steps of chlorophyll biosynthesis were significantly downregulated in T5 vs. T9. In addition, glutamyl-tRNA synthetase (gltX) and oxygen-dependent protoporphyrinogen oxidase (hemL), which are involved in the first step of the process, were downregulated in T10 vs. T11. The downregulation of these proteins was consistent with the circadian biosynthesis of chlorophyll. However, in T9 vs. T10, five proteins—gltX, hemL, magnesium chelatase subunit I (chlI), por and zeaxanthin epoxidase—were significantly upregulated, indicating that photosynthetic pigment biosynthesis was enhanced during this period. Given the previous finding that chlorophyll biosynthesis is a light-induced process, the enhancement of photosynthetic pigment biosynthesis during the dark period is abnormal. Considering the sharp increase in toxin content during this period, there may exist an association between PST production and photosynthetic pigment biosynthesis.

In previous studies, PST biosynthesis has been proposed to be closely associated with chloroplast biosynthesis. PSTs and chlorophyll are thought to share the same polyketide precursor [40]. Chlorophyll formation starts from the synthesis of ALA, where gltX and hemL are the two enzymes that catalyze the conversion of glutamate to ALA [33,34]. Therefore, glutamate provides a carbon skeleton for chlorophyll, and upregulation of gltX and hemL in T9 vs. T10 suggested that utilization of carbon from glutamate to synthesize chlorophyll was enhanced during the toxin production period. For PST production, the carbon skeleton of PSTs originates from arginine, which is synthesized from glutamate [3,31]. Therefore, glutamate, instead of some polyketides, was the common substrate for both chlorophyll biosynthesis and PST production (Figure 5), and the enhanced biosynthesis of glutamate provided sufficient substrate and intermediate for photosynthetic pigment biosynthesis, PST production and protein translation. This close association between photosynthetic pigment biosynthesis and PST production was also observed in our previous study [24]. Because photosynthetic pigments, PSTs and proteins are all nitrogen-enriched macromolecules in toxigenic dinoflagellates and because alterations in these macromolecules always occur simultaneously [17,41], an ecological role of PSTs as intracellular nitrogen stores is plausible in dinoflagellates [42,43].

## 4. Materials and Methods

### 4.1. Culture Conditions and Sample Collection

Unialgal cultures of ACHK-T were provided by the Collection Center of Marine Algae, Xiamen University, China. Cultures of ACHK-T were grown in *K*-medium [44] at 20 °C. Irradiance of approximately 100 μE·m^−2^·s^−1^ was provided using cool white fluorescent bulbs under a 14:10 h light: dark photoperiod.

Synchronization and sampling of ACHK-T cells were conducted using a previously reported method [24,45]. Briefly, the algal cells floating in the upper layer of the culture flask were inoculated into fresh medium with algal cell suspension:medium = 1:3 every 4 days for a month. Then, the synchronized cells were finally transferred to 3 5-L flasks filled with 4 L of *K*-medium and grown for 2 days. Sample collection was started at 08:00 (T1) and carried out every 2 h for 24 h. One milliliter of each culture was collected and fixed with Lugol’s solution for cell counting; 50 mL of culture was collected and the cell pellets were resuspended in 1 mL of 70% ethanol for flow cytometric (FCM) analysis; and 50 mL of culture was collected by centrifugation and stored at −20 °C for toxin analysis. For proteomic analysis, cells were harvested at 16:00 (T5), 00:00 (T9), 02:00 (T10) and 04:00 (T11) based on the toxin analysis result. T5 was the middle time point, at which the toxin content increased slowly; T9 was the time point at which the toxin content started to increase rapidly; and T10 and T11 were the transition and termination time points of this rapid increase. For each time point, 500 mL of each culture (approximately 10^6^ cells) was collected and the cell pellets were resuspended in 1 mL of TRIzol reagent (Invitrogen, Carlsbad, CA, USA), frozen in liquid nitrogen and stored at −80 °C.

### 4.2. Toxin Analysis

PST analysis was conducted by following our previous study [46]. Cell pellets were suspended in 0.5 mL of 50 mM acetic acid and homogenized with sonication. Then, the supernatant was collected by centrifugation at 10,000 ×g for 30 min and filtered with 0.22-μm-pore filter membranes (Millipore). The toxin analysis was carried out using HPLC with post column derivatization by an Intersil C8-5 column. Toxin standards were purchased from the National Research Council, Canada and the concentration of PSTs was determined by comparing the peak area of the sample with that of the toxin standards.

### 4.3. Flow Cytometric Analysis

FCM analysis was conducted by following the previous study [22]. The FCM sample was centrifuged at 10,000× *g* for 5 min and washed twice with phosphate-buffered saline (1× PBS, pH 8.0). Then the sample was suspended in 100 μL of RNase A, incubated at room temperature for 10 min. After that, the sample was suspended in 1 mL of 1× PBS containing 2.5 mg·mL^−1^ propidium iodide and stained for 1 h at 37 °C. The DNA content of stained cells was analyzed using an Epics XL flow cytometer (Beckman Coulter, Miami, FL, USA), and the cell cycle was determined based on the histograms of the relative DNA content, which was analyzed using MultiCycle software (Version 4.0, Phoenix Flow Systems, San Diego, CA, USA).

### 4.4. Protein Preparation

The protein samples were homogenized with sonication, and protein was extracted using TRIzol reagent (Invitrogen, Carlsbad, CA, USA), chloroform and ethanol, and precipitated using isopropanol. Then, the protein pellets were washed with 95% ethanol and air-dried [46]. After that, 100 μL of rehydration buffer was added to dissolve the pellets. Then, the proteins were reduced with 10 mM DTT at 56 °C for 1 h, alkylated with 55 mM iodacetamide and precipitated with 4 volumes of acetone. After air-drying, 500 μL of 0.5 M tetraethyl-ammonium bromide (TEAB) (Applied Biosystems, Milan, Italy) was added to dissolve the pellets and the protein concentration was quantified with the Bradford assay, using bovine serum albumin (BSA) as a standard.

### 4.5. Peptide Labeling

Protein samples from 4 time points were compared using 2 biological replicates each. A total of 100 μg protein from each sample was digested using Trypsin Gold (Promega, Madison, WI, USA) with protein:trypsin = 30:1 at 37 °C for 16 h. After drying by vacuum centrifugation, the trypsin-digested samples were reconstituted in 0.5 M TEAB and labeled with 8-plex iTRAQ reagent (Applied Biosystems, Foster City, CA, USA) according to the manufacturer’s instructions. Samples were labeled with the iTRAQ tags as follows: T5-1 (119 tag), T5-2 (121 tag), T9-1 (117 tag), T9-2 (118 tag), T10-1 (115 tag), T10-2 (116 tag), T11-1 (113 tag) and T11-2 (114 tag). After 2 h of incubation at room temperature, the labeled samples were pooled and dried using vacuum centrifugation.

### 4.6. Cation Exchange Fractionation

The cation exchange fractionation was performed using an LC-20AB HPLC pump system (Shimadzu, Kyoto, Japan). The labeled peptide mixtures were reconstituted with 4 mL of buffer A (25 mM NaH_2_PO_4_ in 25% ACN, pH 2.7) and loaded onto a column containing 5-μm particles (Phenomenex). The peptides were eluted at a flow rate of 1 mL·min^−1^ with the following gradient: buffer A for 10 min, 5–60% buffer B (25 mM NaH_2_PO_4_, 1 M KCl in 25% ACN, pH 2.7) for 27 min, and 60–100% buffer B for 1 min. The system was then maintained at 100% buffer B for 1 min before equilibrating with buffer A for 10 min prior to the next injection. Elution was monitored by measuring the absorbance at 214 nm, and fractions were collected every 1 min. The eluted peptides were pooled into 20 fractions, desalted with a Strata X C18 column (Phenomenex, Torrance, CA, USA) and vacuum-dried.

### 4.7. LC-MS/MS Analysis

All peptide samples were separated on a LC-20AD nanoHPLC system (Shimadzu, Kyoto, Japan) and analyzed on a Q-EXACTIVE system (Thermo Fisher Scientific, San Jose, CA, USA). Each fraction was resuspended in buffer C (2% ACN, 0.1% formic acid) and the final concentration of peptide was about 0.5 μg·μL^−1^ on average. The samples were loaded at 8 μL·min^−1^ for 4 min, and then, a 44-min gradient was run at 300 nL·min^−1^ starting from 2% to 35% buffer D (98% ACN, 0.1% formic acid), followed by a 2-min linear gradient to 80% and maintenance at 80% for 4 min, finally returning to 5% in 1 min.

Peptides were selected for MS/MS using the high-energy collision dissociation (HCD) operating mode with a normalized collision energy setting of 27.0. Survey scans were acquired at a resolution of 70,000 and resolution for HCD spectra was set to 17,500. A data-dependent procedure that alternated between 1 MS scan followed by 15 MS/MS scans was applied for the 15 most abundant precursor ions that were above a threshold ion count of 20,000 in the MS survey scan with a subsequent dynamic exclusion duration of 15 s. The electrospray voltage applied was 1.6 kV. Automatic gain control was used to optimize the generated spectra. For MS scans, the m/z scan range was 350 to 2000 Da. For MS2 scans, the m/z scan range was 100–1800.

### 4.8. Bioinformatics Analysis

MASCOT genetic format files that were obtained by using Proteome Discoverer 1.2 (PD 1.2, Thermo Fisher Scientific, San Jose, CA, USA) to covert raw data files were searched, and protein identification was performed using the MASCOT search engine (Matrix Science, version 2.3.02) against database containing amino acid sequences translated from unigenes in two previous projects [15,23].

For protein identification, a mass tolerance of 0.1 Da was permitted for fragmented ions and 0.05 Da for peptide masses, with allowance for one missed cleavage in the trypsin digests. The charge states of peptides were set to 2+ and 3+. iTRAQ labeling and carbamidomethylation were defined as fixed modifications, and Gln->pyro-Glu (N-term Q), oxidation (M), and dehydration (NQ) were the potential variable modifications. Specifically, an automatic decoy database search was performed in MASCOT by choosing the decoy checkbox in which a random sequence of database is generated and tested for raw spectra as well as the real database. Peptides with significance scores (≥20) at the 99% confidence interval were counted as being identified, and each identified protein contained at least one unique peptide.

For protein quantitation, it was required that a protein contained at least two unique peptides. The quantitative ratios were weighted and normalized using the median ratio in MASCOT. Proteins were subjected to three pairwise comparisons: T5 vs. T9, T9 vs. T10 and T10 vs. T11. A protein was considered to be differentially expressed if it contained at least two unique peptides, had similar and significant ratios ≥1.2 (upregulated) or ≤0.83 (downregulated) in two replicates [7].

Functional annotation of the proteins was conducted using the Blast2GO program against the KEGG and NCBInr databases.

### 4.9. Identification of Toxin-Related Proteins

To identify proteins associated with PST biosynthesis, a BLAST comparison was performed of the toxin-related protein sequences from the cyanobacterium *Cylindrospermopsis raciborskii* T3 [3] against the proteins identified. All hits with e-value ≤ 1 × 10^−5^ were retrieved as confident identifications.

## 5. Conclusions

This study investigated PST production in toxigenic *A. catenella* during the cell cycle and compared the global protein expression profiles at four different stages of toxin biosynthesis using an iTRAQ-based quantitative proteomic approach. Toxin content increased rapidly in the late G1 phase and decreased sharply when the cells entered the G2/M phase. SxtA and sxtI were identified in dinoflagellates for the first time, and the expression of sxtI and ompR was consistent with the toxin biosynthesis pattern. The regulation of toxin biosynthesis in dinoflagellates was similar to that in cyanobacteria, and some steps of the PST biosynthesis process might be regulated post-translationally. Moreover, PST production was modulated by protein translation and photosynthetic pigment biosynthesis via the common substrate glutamate. Overall, our study provided new insights into PST biosynthesis of dinoflagellates and clarified the complex relationship between the cell cycle and PST production.

## Figures and Tables

**Table 1:** Identification and quantitation of toxin-related proteins in *Alexandrium catenella*.

Protein	Top Hit Protein	T5 vs. T9		T9 vs. T10		T10 vs. T11	
		Ratio 1	Ratio 2	Ratio 1	Ratio 2	Ratio 1	Ratio 2
sxtA	CL2951.Contig2_All	-	-	-	-	-	-
sxtG	CL1611.Contig1_All	0.86	1.09	1.12	0.87	0.95	0.86
sxtH	comp55174_c0_orf1	0.76	0.90	1.14	0.80	0.88	1.23
sxtI	comp65949_c0_orf1	0.92	1.49 *	1.57	0.92	0.71 *	0.81 *
sxtO	CL4284.Contig1_All	1.02	1.07	0.92	1.00	1.07	0.93
sxtT	comp55174_c0_orf1	0.76	0.90	1.14	0.80	0.88	1.23
sxtU	Unigene83583_All	1.22	1.53	1.13	0.83	0.94	1.23
sxtZ	comp61883_c0_orf1	0.87	1.14 *	1.19 *	1.04	1.06	1.02
ompR	comp17794_c0_orf1	1.02	0.97	1.10	1.29 *	0.81 *	0.77 *

-: unquantifiable; *: *p*-value ≤ 0.05.

## Supplementary Materials

The following are available online at http://www.mdpi.com/1660-3397/16/12/491/s1. Supplementary Table S1: The identification, quantitation and annotation of the 7232 proteins. Supplementary Table S2: Functional classifications of the differentially expressed proteins.
